# Effect of Polymerization Time on the Binding Properties of Ciprofloxacin-Imprinted nanoMIPs Prepared by Solid-Phase Synthesis

**DOI:** 10.3390/polym13162656

**Published:** 2021-08-10

**Authors:** Matteo Chiarello, Laura Anfossi, Simone Cavalera, Fabio Di Nardo, Fiora Artusio, Roberto Pisano, Claudio Baggiani

**Affiliations:** 1 Department of Chemistry, University of Torino, 10125 Torino, Italy; matteo.chiarello@unito.it (M.C.); laura.anfossi@unito.it (L.A.); simone.cavalera@unito.it (S.C.); fabio.dinardo@unito.it (F.D.N.); 2 Department of Applied Science and Technology, Polytechnic University of Torino, 10125 Torino, Italy; fiora.artusio@polito.it (F.A.); roberto.pisano@polito.it (R.P.)

**Keywords:** molecularly imprinted polymer, solid-phase synthesis, nanoparticles, ciprofloxacin, binding equilibrium, binding kinetics, binding selectivity

## Abstract

An innovative approach to imprinted nanoparticles (nanoMIPs) is represented by solid-phase synthesis. Since the polymeric chains grow over time and rearrange themselves around the template, the binding properties of nanoMIPs could depend on the polymerization time. Here we present an explorative study about the effect of different polymerization times on the binding properties of ciprofloxacin-imprinted nanoMIPs. The binding properties towards ciprofloxacin were studied by measuring the binding affinity constants (*K_eq_*) and the kinetic rate constants (k_d_, k_a_). Furthermore, selectivity and nonspecific binding were valued by measuring the rebinding of levofloxacin onto ciprofloxacin-imprinted nanoMIPs and ciprofloxacin onto diclofenac-imprinted nanoMIPs, respectively. The results show that different polymerization times produce nanoMIPs with different binding properties: short polymerization times (15 min) produced nanoMIPs with high binding affinity but low selectivity (*K_eq_* > 10^7^ mol L^−1^, α ≈ 1); medium polymerization times (30 min–2 h) produced nanoMIPs with high binding affinity and selectivity (*K_eq_* ≥ 10^6^ mol L^−1^, α < 1); and long polymerization times (>2 h) produced nanoMIPs with low binding affinity, fast dissociation kinetics and low selectivity (*K_eq_* ≤ 10^6^ mol L^−1^, k_dis_ > 0.2 min^−1^, α ≈ 1). The results can be explained as the combined effect of rearrangement and progressive stiffening of the polymer chains around the template molecules.

## 1. Introduction

**Scheme 1:**
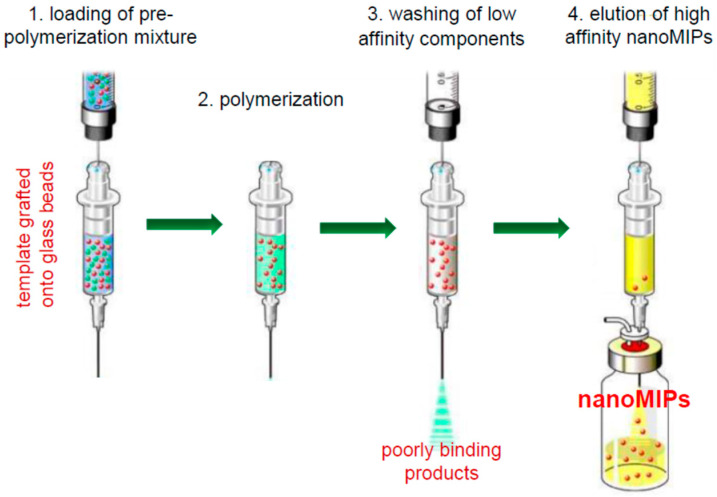
Representation of the solid-phase synthesis method.

Molecularly imprinted nanoparticles (nanoMIPs) present several advantages with respect to bulk imprinted materials, but, when prepared by traditional methods, their usefulness is limited as the approaches are costly or require complex optimization steps, while the purification from template molecules is challenging [1,2,3]. Solid-phase synthesis of imprinted nanoparticles (nanoMIPs) represents an innovative approach to nanoMIPs [4,5]. The polymerization process, illustrated in Scheme 1, takes place in the interstitial space between nonporous glass beads grafted with template molecules. Once the polymerization process ends, unreacted monomers, polymerization by-products and low-affinity polymers can be washed away, while the high-affinity nanoparticles bind strongly enough to be retained by the solid phase. NanoMIPs are subsequently recovered by washing the solid phase with a solution capable of breaking the noncovalent molecular interactions.

This approach presents several practical advantages over traditional solution synthesis techniques: The bleeding effect due to residual template molecules in the imprinted polymer is avoided as the template is covalently grafted onto the solid phase [6]. Grafted templates do not need to be soluble in the polymerization solvent, thus eliminating any issue about solvent–template compatibility [7]. Solid phase can be reused many times, allowing the convenient use of expensive molecules [8]. Toxic or harmful templates are confined on the surface of the beads, thus avoiding any health risks during synthesis and successive use of the imprinted nanoMIPs [9]. The preparation of nanoMIPs by solid-phase synthesis has proven to be equally suitable for small molecules [10,11,12], peptides and proteins [13,14], living cells [15] or viruses [16]. Furthermore, it seems to be very versatile, as the experimental conditions necessary for a successful imprinting process can be changed according to current needs in a more flexible way than the solution synthesis technique [15].

Currently, few published data are available about the effect of the polymerization time on the binding properties of nanoMIPs [4,17,18]. In fact, available experimental protocols provide for very short reaction times—from few seconds to tens of minutes—when using the UV-initiated polymerization, or longer times when using the persulfate/TEMED initiator system, but we have to keep in mind that such polymerization times are intended as reasonable temporal intervals within which polymerization occurs but there is still no formation of insoluble polymer. Consequently, it could be interesting to check whether the polymerization time has an effect on the binding properties of nanoMIPs.

Here we present an explorative study about the effect of different polymerization times—ranging from 15 min to 5 h—on the binding properties of ciprofloxacin-imprinted nanoMIPs. This template was chosen as a system model as imprinted nanoparticles have been extensively reported in the literature as synthetic receptors in sensing devices [19,20] and solid-phase extraction [21]. Moreover, ciprofloxacin is strongly fluorescent, so it can be detected at very low concentrations by HPLC, making it possible to determine the binding isotherms in conditions of high dilution required by the equilibrium constants typical of nanoMIPs prepared by solid-phase synthesis [22]. The binding properties were studied by partition equilibrium and rebind kinetic experiments to measure the binding affinity and the kinetic rate constants. Furthermore, selectivity and nonspecific binding were valued by measuring the rebinding of levofloxacin onto ciprofloxacin-imprinted nanoMIPs and ciprofloxacin onto diclofenac-imprinted nanoMIPs, respectively.

## 2. Materials and Methods

### 2.1. Materials

Glass beads (Spheriglass-2429, 70–100 μm average particle size, Potters, UK) were aminated as previously reported [22]. Acrylic acid (AA), 3-(aminopropyl) trimethoxysilane (APTMS), ciprofloxacin (CIP), *N*,*N*-dimethylaminopyridine (DMAP), 1-ethyl-3-(3-dimethylaminopropyl)carbodiimide hydrochloride (EDC), hexamethyldisilazane (HMDS), *N*-hydroxysuccinimide (NHS), *N*-isopropylacrylamide (NIPAm), levofloxacin (LEV), *N*,*N*′-methylene-bis-acrylamide (BIS), morpholinethanesulfonic acid (sodium salt, MES), succinic anhydride, *N*-tertbutylacrylamide (TBAm), ammonium persulfate (APS), *N*,*N*,*N*′,*N*′-tetramethylethylenediamine (TEMED) were obtained from Sigma-Merck (Milan, Italy). Solvents and all other chemicals were purchased from Sigma-Merck (Milan, Italy). All the solvents were of HPLC grade, whereas all chemicals were of analytical grade. The water used was ultrapurified in Purelab Prima System from Elga (Marlow, UK). Antibiotic stock solutions were prepared by dissolving 25 mg of the substance in 25 mL of water/methanol 1 + 1 (*v/v*) then stored in the dark at −20 °C.

### 2.2. Synthesis of Ciprofloxacin Hemisuccinamide

**Scheme 2:**
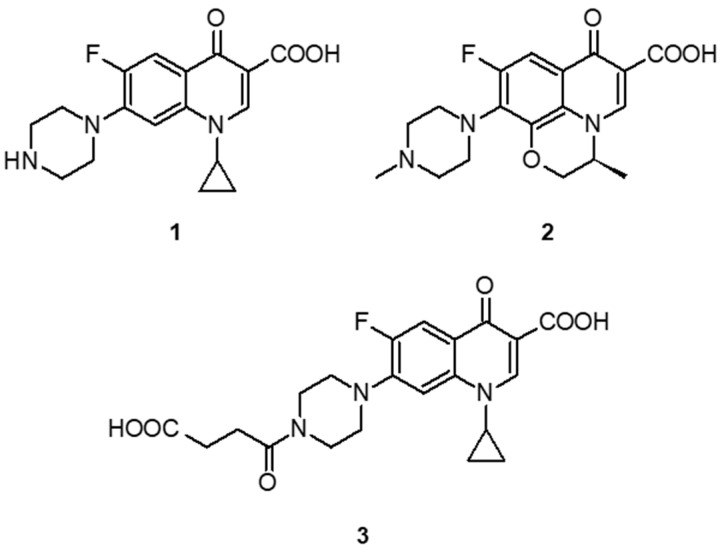
Ciprofloxacin (**1**), levofloxacin (**2**), ciprofloxacin hemisuccinamide (**3**).

The template molecule, ciprofloxacin hemisuccinamide (CIP-HS, Scheme 2), was synthesized according to a modification of the procedure given by Noël et al. [23]. Succinic anhydride (31 mg, 0.31 mmol) was added to a suspension of CIP (100 mg, 0.30 mmol) in DMSO (2.0 mL) containing a catalytical amount of DMAP. The reaction mixture was stirred under nitrogen overnight at 80 °C and cooled down to room temperature. The resulting precipitate was filtered, washed successively with water and diethylether and dried under reduced pressure. The expected hemisuccinamide was isolated as a fluffy white powder (98 mg, yield 75%), deemed pure by MS-HPLC (ESI: *m/z* 431.1 [MH+]).

### 2.3. Synthesis of nanoMIPs

The template CIP-HS was grafted onto the aminated glass beads in accordance with the protocol previously reported [22]. The polymerization mixtures were prepared modifying the general protocol reported in the literature [15] and adjusting the dilution of monomers to avoid formation of unwanted lumps of polymer. A prepolymerization mixture (molar ratio BIS:AA:NIPAM:TBAm = 2:20:30:48) was made in 25 mL of ultrapure water by mixing 1 mg of BIS (0.0065 mmol), 4.7 mg of AA (0.065 mmol), 11 mg of NIPAm (0.097 mmol) and 19.8 mg of TBAm (0.156 mmol, dissolved in 0.5 mL of ethanol). Then, 5 mL of the mixture was added to 50 mL polypropylene SPE cartridges containing 2.5 g of functionalized glass beads. The cartridges were purged with nitrogen for 5 min; 3 μL of TEMED and 100 μL of 30 mg mL^−1^ aqueous solution of APS were added; and the polymerization was carried out at room temperature for 15, 30, 45, 60, 120, 180 and 300 min in a roller-equipped incubator. The supernatant was drained by vacuum aspiration, the dry cartridges were cooled to 4 °C and polymerization by-products and low-affinity nanoMIPs were washed with 10 × 2 mL of ice-cold water. High-affinity nanoMIPs were collected by eluting the cartridges with 5 × 2 mL of hot water. The eluates were lyophilized, weighed and stored at 4 °C. Size distribution and polydispersity index were determined as previously reported [22].

Nonimprinted polymers (nanoNIPs) were prepared in the same experimental conditions in terms of composition of the polymerization mixture and polymerization time, but using glass beads functionalized with diclofenac as solid phase [18].

NanoMIPs and nanoNIPs were grafted onto aminated glass beads in accordance with the protocol previously reported [22].

### 2.4. Determination of Binding Properties

To measure equilibrium binding isotherms and kinetics, unbound fractions of fluoroquinolones were measured by reverse-phase HPLC analysis with fluorescence detection, in accordance with previous literature [22]. Each experimental point was assessed as the average of three repeated measures, and binding parameters were calculated in accordance with Langmuir binding isotherm and first-order kinetic models by nonlinear least square fitting.

Binding selectivity, *α*, was calculated as follows:

(1) $$\alpha =\frac{K_{eq\left(LEV\right)}}{K_{eq\left(CIP\right)}}$$

where *K_eq (LEV)_* and *K_eq (CIP)_* are the equilibrium binding constants measured for levofloxacin and ciprofloxacin, respectively.

## 3. Results

To study the effect of the duration of the polymerization process, we considered ciprofloxacin-imprinted nanoMIPs prepared by persulfate/TEMED-induced radical polymerization in water at room temperature. We have recently described these nanoparticles [22], reporting their good binding properties towards fluoroquinolones. Here, we considered polymerization times ranging from 15 min to 5 h. Under all the experimental conditions, the solid-phase synthesis produced nanoMIPs fully soluble in water, resulting in transparent and colorless solutions, without any perceivable turbidity. Yields calculated with respect to the amount of monomers in the polymerization mixtures were 1.0 mg (15%) for 15 min, 1.4 mg (21%) for 30 min, 1.1 mg (17%) for 45 min, 1.8 mg (28%) for 1 h, 4.0 mg (61%) for 2 h, 1.2 mg (18%) for 3 h and 3.5 mg (54%) for 5 h.

### 3.1. Dynamic Light Scattering

Dynamic light scattering measurements performed on nanoMIPs are reported in Table 1. NanoMIPs prepared with a polymerization time of 15 min are characterized by a very small mean diameter (12 ± 5 nm) and a relatively high polydispersity index of 0.44. Instead, nanoMIPs prepared with longer polymerization times show particles whose diameters are of the order of magnitude of hundreds of nanometers and values for the polydispersity index slightly lower and substantially constant. A progressive increase in the average diameter is observed with the polymerization time, indicating that the formation process of the nanoparticles is plausibly independent of the polymerization time for times greater than 15 min.

### 3.2. Determination of Binding Affinity

To correctly evaluate the binding properties of nanoMIPs towards ciprofloxacin, the measurements of equilibrium binding isotherms and association kinetics require fast separation between free and bound ligand. As ultrafiltration or dialysis are quite slow, we chose to support the nanoMIPs on the same glass beads used for their synthesis in order to easily separate by filtration the grafted beads—carrying the bound ligand—from the solution containing the free ligand. Preliminary experiments showed that bare glass beads and HDMS-silanized beads were unable to bind ciprofloxacin in water; therefore, it is reasonable to assume that the existence of binding between ciprofloxacin and solid phase can be attributed to the interaction with nanoMIPs.

The binding parameters obtained from binding isotherm measured for ciprofloxacin and levofloxacin (Figure S1) are reported in Table 2. They confirm that nanoMIPs prepared by solid-phase synthesis strongly bind the template ciprofloxacin and, to a lesser extent, the related fluoroquinolone levofloxacin with equilibrium binding constants (*K_eq_*) in the range 10^6^–10^7^ L mol^−1^, with values progressively decreasing when polymerization time increases. In contrast, the binding of ciprofloxacin to diclofenac-imprinted nanoMIPs (Figure S2)—which can then be regarded as a measure of nonspecific binding—is always low and essentially constant. A statistical comparison (*t*-test: α = 0.05, *n* = 10, *t* < 2.101) of the equilibrium binding constants for ciprofloxacin between nanoMIPs and nanoNIPs shows that when nanoMIPs are polymerized for times longer than 2 h, there is no difference with respect to nanoNIPs.

**Figure 1:**
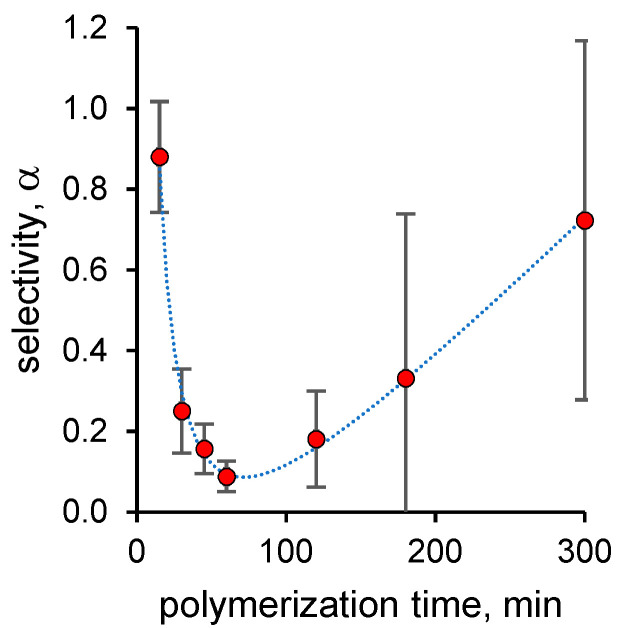
Binding selectivity of ciprofloxacin-imprinted nanoMIPs measured by comparing equilibrium binding constants for levofloxacin and ciprofloxacin. Error bars indicate 1 standard error unit.

Concerning selectivity, as reported in Figure 1, nanoMIPs polymerized for very short times are not able to discriminate between ciprofloxacin and levofloxacin, with a complete lack of selectivity. As the polymerization time increases, the selectivity improves markedly, reaching α ≈ 0.1 at 1 h and then worsening again for longer polymerization times. To evaluate this trend properly, it is necessary to consider that the values of the equilibrium binding constants for both ligands are high but statistically indistinguishable for short polymerization times; therefore, their numerical relationship must be unitary. On the contrary, for longer polymerization times, these values are markedly different between ciprofloxacin and levofloxacin. Indeed, those for levofloxacin cannot be distinguished by nonspecific binding, while those for ciprofloxacin become so only for long polymerization times. Consequently, for intermediate times, a significant selectivity with α values much less than 1 but with a progressive tendency to rise is seen, while for longer times, the lack of selectivity is a consequence of a binding behavior of nanoMIPs indistinguishable from nanoNIPs for both the ligands.

### 3.3. Determination of Binding Kinetics

Equilibrium binding constants (*K_eq_*) measured for ciprofloxacin can be broken down into dissociation (k_dis_) and association (k_ass_) kinetic rate constants, measuring k_ass_ (Figure S3) and calculating k_dis_ from the relationship *K_eq_* = k_ass_/k_dis_. It may therefore be interesting to examine the values of these rate constants, reported in Table 3, in relation to the polymerization times.

**Figure 2:**
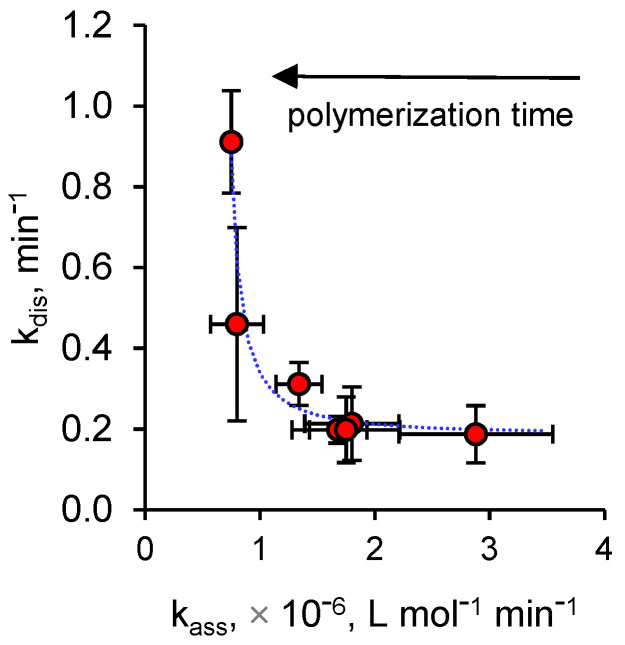
Dissociation rate constants (k_dis_) vs. association rate constants (k_ass_) plot for ciprofloxacin on ciprofloxacin-nanoMIPs. Error bars indicate 1 standard error unit.

As reported in Figure 2, in the whole range of polymerization times considered in this work, both dissociation and association rate constants change in a limited range (k_dis_: 0.19–0.91 min^−1^; k_ass_: 0.75−2.88 × 10^6^ L mol^−1^ min^−1^), but considering only the nanoMIPs that show a greater affinity for ciprofloxacin in comparison with nanoMIPs (15 min–1 h), it can be observed that the dissociation rate constant remains practically unchanged with a mean value of 0.2 min^−1^. It follows that the affinity of ciprofloxacin for the imprinted binding sites mainly depends on the values of association rate constant, values which gradually decrease as the polymerization time increases. On the contrary, poorly binding nanoMIPs obtained with longer polymerization times show totally different kinetic behaviors. In fact, while association rate constants are low and substantially constant, dissociation rate constants are high and proportional to the polymerization time.

## 4. Discussion

It has recently been shown that the composition of the polymerization mixture—very poor in cross-linker component (2% total moles in this work)—in the solid-phase synthesis technique is such that the concept about the formation of a rigid and well-defined binding site within a highly cross-linked network of polymeric chains must be discarded. Alternatively, a model has been proposed where the molecular imprinting of nanoparticles is the result of the dynamic interaction between the template molecules grafted onto the surface of the solid phase and the lightly cross-linked chains being formed at the interface between the solution and the glass surface [24].

Within the framework of this model, as the polymeric chains grow progressively over time and rearrange themselves around the immobilized template, the binding properties of nanoMIPs depend not only on the classic parameters typical of molecular imprinting, such as the ability of functional monomers to interact with the template, but also on the duration of the polymerization process, i.e., the actual dimensions of the nanopolymers.

The experimental results reported in this work confirm that the polymerization time in the solid-phase polymerization method has different effects on the binding behavior of the resulting nanoMIPs according to the temporal extent of the polymerization process: (i) short polymerization times, with nanoparticles characterized by high binding affinity but low selectivity (*K_eq_* > 10^7^ mol L^−1^, α ≈ 1); (ii) medium polymerization times, with nanoparticles characterized by high binding affinity and selectivity (*K_eq_* ≥ 10^6^ mol L^−1^, α < 1); and (iii) long polymerization times, with nanoparticles characterized by low binding affinity, faster dissociation kinetics and low selectivity (*K_eq_* ≤ 10^6^ mol L^−1^, k_dis_ > 0.2 min^−1^, α ≈ 1).

To try to evaluate this complex behavior, it is necessary to consider that the nanoparticles continue to grow during the polymerization process, both when they are bound to the solid phase and when they are free in solution. In the early stage of the polymerization, it is plausible that the conformation of the growing polymeric chains is very flexible and able to rapidly rearrange and maximize the interaction with the template on the solid phase. In these conditions, there are no binding sites with a stiff and defined structure yet, even if, once dissociated, nanoparticles are able to maintain—at least in part—the binding conformation but with limited selectivity towards molecules other than the template. As a consequence, nanoMIPs prepared in very short polymerization time show a type-i binding behavior, with high binding affinity and fast association kinetics but poor or absent selectivity.

When the polymerization process continues for longer times, nanoparticles become larger and more structured, with restrained and intertwined chain conformation, leading to binding sites that are well defined but stiffer and—after dissociation from the solid phase—less accessible to ligands. In this condition, nanoMIPs show a type-ii binding behavior: the association rate constant and the binding affinity decrease but selectivity increases.

Further prolonging the polymerization time causes further growth of the nanoparticles. Under these conditions, the polymer structure becomes more and more rigid, sterically hindering the binding sites to the point that they cannot effectively bind the ligands during the rebinding process. In this condition, nanoMIPs show a type-iii binding behavior: the binding properties coincide with those of nonimprinted nanoparticles, no longer depending on the presence of accessible binding sites but only on the presence of randomly dispersed functional groups on the surface of the nanoparticles.

## 5. Conclusions

The results reported in this work show that polymerization time plays a pivotal role in determining the binding properties of nanoMIPs prepared by persulfate/TEMED-induced radical polymerization. It is reasonable to assume that, since the growth rate of the nanoparticles certainly depends on many variables such as the chemical properties of the monomers, the solvent, the polymerization temperature and the type of radical initialization, the results reported here are to be considered representative of this system in particular. However, there is no reason to believe that the overall behavior implied by the experimental results reported here cannot be extended to different polymerization mixtures and experimental conditions and therefore be of general validity for nanoMIPs prepared by solid-phase synthesis. Finally, we conclude by stressing that the control of the polymerization time allows calibrating not only the binding capacity of the nanoMIPs towards the template, both in terms of affinity and association kinetics, but also the selectivity towards related molecules. This result seems to us particularly interesting for future applications that will require nanoMIPs with a high capacity to discriminate between ligands with similar molecular structures.

## Figures and Tables

**Table 1:** Dynamic light scattering results for ciprofloxacin-imprinted nanoMIPs.

Polymerization Time	Diameter (nm)	Polydispersity Index
15 min	12 ± 5	0.44
30 min	120 ± 41	0.34
45 min	165 ± 42	0.26
1 h	154 ± 37	0.24
2 h	234 ± 84	0.36
3 h	236 ± 87	0.37
5 h	268 ± 94	0.35

**Table 2:** Equilibrium binding constants (±1 standard error unit) measured for ciprofloxacin and levofloxacin on ciprofloxacin-imprinted nanoMIPs and ciprofloxacin on diclofenac-imprinted nanoMIPs (nanoNIPs). Underlined values: statistically indistinguishable from the corresponding results obtained on nanoNIPs.

Polymerization Time	CIP on nanoMIPs (L mol^−1^ × 10^−6^)	LEV on nanoMIPs (L mol^−1^ × 10^−6^)	CIP on nanoNIPs (L mol^−1^ × 10^−6^)
15 min	15.39 ± 2.14	13.54 ± 0.95	1.96 ± 0.38
30 min	8.48 ± 0.81	2.12 ± 0.86	2.25 ± 0.68
45 min	8.44 ± 1.75	1.32 ± 0.44	0.57 ± 0.22
1 h	8.84 ± 1.65	0.78 ± 0.30	0.82 ± 0.44
2 h	4.30 ± 0.60	0.77 ± 0.50	1.14 ± 0.21
3 h	1.74 ± 0.87	0.58 ± 0.65	1.21 ± 0.86
5 h	0.82 ± 0.33	0.60 ± 0.28	~1 ^1^

^1^ Lack of fit, estimated only.

**Table 3:** Association and dissociation rate constants (±1 standard error unit) measured for ciprofloxacin on ciprofloxacin-imprinted nanoMIPs.

Polymerization Time	k_ass_ (L mol^−1^ min^−1^ × 10^−6^)	k_dis_ (min^−1^)
15 min	2.88 ± 0.67	0.19 ± 0.07
30 min	1.68 ± 0.25	0.20 ± 0.03
45 min	1.80 ± 0.41	0.21 ± 0.09
1 h	1.75 ± 0.47	0.20 ± 0.08
2 h	1.34 ± 0.20	0.31 ± 0.05
3 h	0.80 ± 0.23	0.46 ± 0.24
5 h	0.75 ± 0.04	0.91 ± 0.13

## Data Availability

The raw and processed data required to reproduce these findings are available on request.

## Supplementary Materials

The following are available online at https://www.mdpi.com/article/10.3390/polym13162656/s1, Figure S1: Binding isotherm of ciprofloxacin and levofloxacin for ciprofloxacin-imprinted nanoMIPs, Figure S2: Binding isotherm of ciprofloxacin for diclofenac-imprinted nanoMIPs, Figure S3: Association kinetic plots of ciprofloxacin for ciprofloxacin-imprinted nanoMIPs.
