# Supplementary material for: Effect of Polymerization Time on the Binding Properties of Ciprofloxacin-Imprinted nanoMIPs Prepared by Solid-Phase Synthesis
Source: Polymers (Basel). 2021 Aug 10;13(16):2656. doi: 10.3390/polym13162656 (PMC8398629; doi:10.3390/polym13162656)
Supplement: Supplementary file 1 [file polymers-13-02656-s001.zip › polymers-1321736-supplementary.pdf]

polymers

Article

Effect of Polymerization Time on the Binding Properties of Ciprofloxacin-Imprinted nanoMIPs Prepared by Solid-Phase Synthesis

Matteo Charello [1], Laura Anfossi [1], Simone Cavalera [1], Fabio Di Nardo [1], Fiora Artusio [2], Roberto Pisano [2] and Claudio Baggiani [1,*]

[1] Department of Chemistry, University of Torino, 10125 Torino, Italy; matteo.chiarello@unito.it (M.C.); laura.anfossi@unito.it (L.A.); simone.cavalera@unito.it (S.C.); fabio.dinardo@unito.it (F.D.N.)
[2] Department of Applied Science and Technology, Polytechnic University of Torino, 10125 Torino , Italy; fiora.artusio@polito.it (F.A.); roberto.pisano@polito.it (R.P.)
* Correspondence: claudio.baggiani@unito.it

Citation: Charello, M.; Anfossi, L.; Cavalera, S.; Di Nardo, F.; Artusio, F.; Pisano, R.; Baggiani, C. Effect of Polymerization Time on the Binding Properties of Ciprofloxacin-Imprinted nanoMIPs Prepared by Solid-Phase Synthesis. *Polymers* 2021, *13*, x. https://doi.org/10.3390/xxxxx

Academic Editor: Beom Soo Kim

Received: 15 July 2021
Accepted: 5 August 2021
Published: 10 August 2021

Publisher's Note: MDPI stays neutral with regard to jurisdictional claims in published maps and institutional affiliations.

Copyright: © 2021 by the authors. Licensee MDPI, Basel, Switzerland. This article is an open access article distributed under the terms and conditions of the Creative Commons Attribution (CC BY) license (http://creativecommons.org/licenses/by/4.0/).

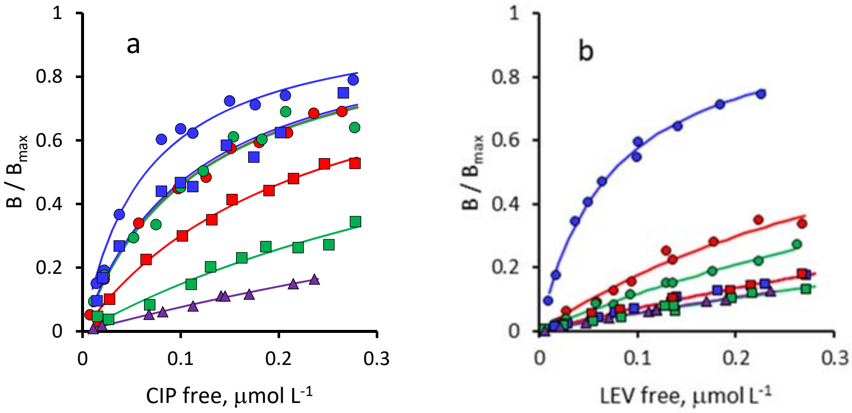

Figure S1. Binding isotherm of (a) ciprofloxacin, (b) levofloxacin for ciprofloxacin-imprinted nanoMIPs. Polymerization times: blue circles, 15 min; red circles, 30 min; green circles, 45 min; blue squares, 1 h; red squares, 2 h; green squares, 3 h; violet triangles, 5 h.

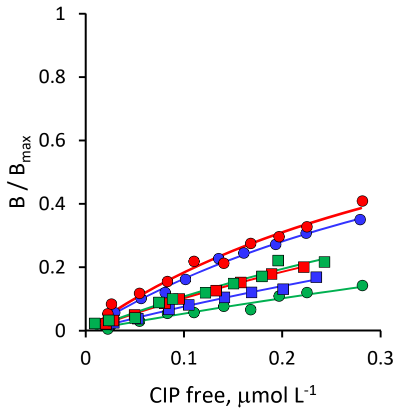

Figure S2. Binding isotherm of ciprofloxacin for diclofenac-imprinted nanoMIPs. Polymerization times: blue circles, 15 min; red circles, 30 min; green circles, 45 min; blue squares, 1 h; red squares, 2 h; green squares, 3 h; violet triangles, 5 h.

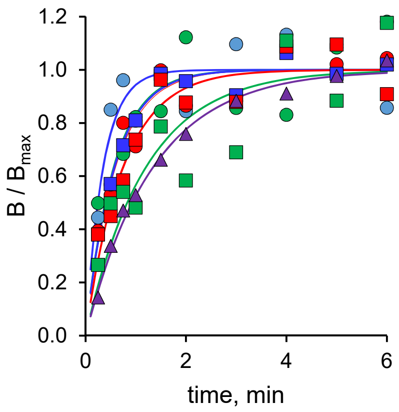

Figure S3. Association kinetic plots of ciprofloxacin for ciprofloxacin-imprinted nanoMIPs. Polymerization times: blue circles, 15 min; red circles, 30 min; green circles, 45 min; blue squares, 1 h; red squares, 2 h; green squares, 3 h; violet triangles, 5 h.
